# Multi-residue analysis of certain lanolin nipple care products for trace contaminants

**DOI:** 10.1186/s13065-023-00919-0

**Published:** 2023-02-27

**Authors:** Katie Bourdillon, Tom McCausland, Morgan McCabe

**Affiliations:** Lansinoh Laboratories, Leeds, UK

**Keywords:** Breastfeeding, Nipple damage, Pesticides, Lanolin, FLA

## Abstract

**Background:**

Topical lanolin is commonly used on nipples to aid breastfeeding success. The raw material undergoes refinement to remove contaminants such as pesticides, which may accumulate from exogenous environmental sources. The level of refinement influences final lanolin purity. For use in nipple creams, a lanolin which complies with a published monograph (either USP or Ph. Eur.) is desirable to ensure a non-toxic product with neutral taste and smell, and low allergenicity.

**Methods:**

The aim of this study was to determine the residual trace pesticide levels and quantify the Free Lanolin Alcohols (FLA) levels in two commercially available lanolin products (HPA LANOLIN (Lansinoh Laboratories Inc.); PURELAN (Medela AG)) and two lanolin ingredients (PHARMALAN PH EU-SO-(RB) and CORONA-8 SO-(RB) (Croda, Goole, UK)) using established validated methods. Test samples were subjected to Gas-Chromatographic and Liquid-Chromatographic analysis to quantify and identify a panel of 178 pesticide residues. FLA levels and the presence of oxidative metabolites were also determined.

**Results:**

The purity of the lanolin ingredients was consistent with expectations based on their level of refinement; lanolin in compliance with the Ph. Eur. monograph demonstrated the highest level of purity. Differences were seen between the lanolin nipple creams tested, in terms of FLA levels and pesticide residue levels. Specifically, the HPA LANOLIN contained an extremely low level of FLA (0.61%), which was fourfold less than in the PURELAN (2.76%). Additionally, the HPA LANOLIN did not contain any detectable pesticide residues. The PURELAN was found to contain a number of pesticide residues, however the detected levels were low and within the permitted limits and so despite their presence, the PURELAN was still compliant with the relevant monographs.

**Conclusions:**

This data reinforces that the purity of monograph compliant Lanolin makes it suitable for use on the nipples of breastfeeding mothers. A higher level of refinement leads to a reduction in pesticide contaminants also reduces FLA levels in the final material, minimizing the risk of allergenicity.

## Background

Human milk is the recommended source of nutrition for the first 6 months of an infant’s life, followed by human milk along with appropriate complementary foods up to two years of age or beyond [1]. However, many new mothers experience cracked, sore or painful nipples particularly in the early stages of breastfeeding which can impact their ability to meet their breastfeeding goals [2–4].

Historically, mothers have been advised to express a small amount of milk onto sore nipples after feeding to manage nipple related issues [5], however a variety of commercially available ointments and creams are also available to help support breastfeeding success. One commonly used solution is purified lanolin. Lanolin is a wax derived from sheep wool and its natural purpose is to coat the wool fibres and skin of the sheep, protecting it from infection and the elements [6]. It is structurally very similar to lipids found within the skin, particularly in the stratum corneum. These lipids contribute to the integrity of the skin barrier [7]. This makes lanolin an excellent moisturizer and emollient, as it forms a stable emulsion with water in the skin to prevent evaporation and retain moisture [8].

The recorded history of people extracting and utilising crude lanolin from sheep’s wool for its emollient properties goes back thousands of years to Ancient Greece [9]. Modern day lanolin undergoes a complex refinement process in order to obtain a highly purified lanolin ingredient from raw wool wax; this is necessary as the raw material may be contaminated with environmental impurities such as pesticide residues, detergents used in the wool scouring process and salts [9]. Pesticides are used to prevent fleece damage by sheep ectoparasites and to protect wool during storage; historically, the most commonly used were organochlorine, organophosphorus and pyrethroid insecticides [10]. Due to the lipophilic nature of these pesticides, they accumulate in wool wax [9] and their environmental persistence means they are still a common contaminant of wool grease to this day.

Removal of these impurities typically requires a multi-stage refining sequence, as described by Clark (1999), who considered the refinement process a combination of technique and science, highlighting that method variability will influence the purity level and properties of the lanolin [9, 11]. Simplistically, the higher the level of refinement, the more pesticide and other contaminants will be removed. Super-refinement also results in a paler and more stable lanolin material (although sometimes lanolin may be artificially lightened through a bleaching step) and odour compounds are also removed. These are desirable product attributes for minimal interference with the intricacies of breastfeeding. Levels of free lanolin alcohols (FLA) also decrease as the level of refinement increases [9]. The refinement process can be costly, both due to the complex methodology and the amount of material lost during processing. The conditions and level of refinement of lanolin can therefore be optimised depending on the final application of the ingredient; in addition to cosmetic and pharmaceutical applications, lower grades of lanolin are used for industrial purposes [11].

When lanolin is to be used as a cosmetic or pharmaceutical ingredient, a lanolin grade which complies to the United States Pharmacopoeia (USP) or European Pharmacopoeia (Ph. Eur.) is desirable [9]. These set limits for acidity, alkalinity, free alcohols, detergents and pesticide residues as well as other parameters relating to general purity [9]. While the USP and Ph. Eur. Standards are similar, there are some key differences in their requirements. For pesticides, the USP monograph for modified lanolin sets a maximum permitted level of 1 part per million (ppm) for any individual specified residue, and no more than 3 ppm total specified residues. The European monograph is stricter, setting a maximum of 0.05 ppm for each specified organochloropesticide, 0.5 ppm for each other specified pesticide, and no more than 1.0 ppm total specified residues. FLA are the component in lanolin which has historically been associated with allergenicity in some users when present at higher concentrations, although it is now acknowledged that the risk of sensitization to lanolin may have been overstated and that a lanolin with <1.5% FLA may be considered truly hypoallergenic [12, 13]. A maximum concentration of FLA of 6.0% is part of the USP, however there is no equivalent maximum threshold in the European standard [14, 15]. In reality, high-grade lanolin often exceeds the specifications outlined in both monographs. This low allergenic profile and extremely pure final material make it particularly useful for the specialised application of nipple care in breastfeeding mothers, where the lanolin does not require removal prior to nursing so may result in incidental ingestion by the infant.

The aim of this study was to determine the residual trace pesticide levels and quantify the FLA levels in two commercially available lanolin products (HPA LANOLIN (Lansinoh Laboratories Inc.); PURELAN (Medela AG)) and two lanolin ingredients (PHARMALAN PH EU-SO-(RB) and CORONA-8 SO-(RB) (Croda) using established methods. This study used validated methods to determine the residual trace pesticide levels and determine the FLA levels in commercially available lanolin products and ingredients. Oxidative stability of the samples, which is critical in determining stability and shelf life of edible fats and oils and is therefore an important quality parameter was also investigated through chemical determinations of peroxide value (PV), anisidine value (AnV) and Acid Value (AV) [16]. These by-products are markers of lanolin rancidity and higher levels can negatively impact the smell and taste of lanolin, which may be distasteful to a nursing infant.

## Methods

Residue analytics was conducted by an accredited independent chemical-analytical laboratory using validated methods. All data was analysed and interpreted by the authors.

### Test materials

Two 100% lanolin products specifically indicated for breastfeeding use; HPA LANOLIN (Lansinoh Laboratories Inc., VA, USA), and PURELAN (Medela AG, Baar, CH) were evaluated, along with two other lanolin ingredients; PHARMALAN PH EU-SO-(RB) (Croda, Goole, UK) and CORONA-8 SO-(RB) (Croda, Goole, UK). Three independent batches of each commercial product were tested, while the lanolin ingredients were tested in singlicate.

### Residue screening

Test samples were screened for trace amounts of 178 different pesticides including organochloropesticide (OCP), pyrethroid insecticide and organophosphoropesticide (OPP) compounds. The screening panel included pesticides listed as reference pesticides in the USP monograph for modified lanolin, and European Pharmacopoeia for wool fat, along with other environmental pesticides known be potential contaminants of lanolin and other anhydrous materials. Tables 1 and 2 outline the full pesticide residue screening panel and the associated limits of quantification. The method for determination of pesticide residues was based on DIN EN 12393, with method development and validation previously published by Cetinkaya and confirmed to conform with SANCO/12495/2011 [17, 18]. Firstly, the sample was melted at 50–70 °C in a water bath and homogenised with a glass rod before a 1 g sample was diluted in up to 10 ml ethyl acetate/cyclohexane (1:1 v/v), homogenised and filtered through a teflon membrane filter. This filtrate was purified by gel permeation chromatography (GPC Autoprep 1002 B, ABC Laboratories Columbia-USA) with test material partitioned into ethyl acetate/cyclohexane (1:1 v/v. elution speed 5 ml/min, injection volume 10 ml) using a 32 cm long gel column packed with 50 g Bio Beads SX-3. After 19 min dump time, collection time followed for 15 min. Finally, the system was washed for 2 min [17]. The purified extract was carefully rotary-evaporated down at a reduced pressure of 50–100 mbar and a water bath temperature of 40–50 °C. Each sample was then made up to a defined volume of 5 ml with methanol and treated in an ultra-sonic bath for 1 min, then filtered through a teflon membrane filter. Additional screening of pesticides utilised the QuEChERS method EN 15662 2008 [19]. Extraction was performed with acetonitrile, followed by freeze out and optional clean up with diamino/C18 dSPE before analysis. The purified solution was analysed by gas chromatography with an electron-capture detector (Carlo Erba HRGC 5300) and a thermoionic detector (Fisons Instruments HRGC Mega 2 Series), followed by confirmation of residue identification and quantification by mass spectrometry (GC–MS/MS (Thermo EVO8000), LC–MS/MS (Absciex, 4000QTRAP) [17].

**Table 1:** Organochloropesticide and Pyrethroid screening panel

Organochloropesticides	LOQ (mg/kg)
Instrumentation: GC-ECD (Carlo Erba HRGC 5300)	
Hexachlorobenzene^a,b^	< 0.01
α-Hexachlorocyclohexane (Alpha-HCH)^a,b^	< 0.01
β-Hexachlorocyclohexane (Beta-HCH)^a b^	< 0.01
Lindane^a,b^	< 0.01
delta-Hexachlorocyclohexane (Delta-HCH)^b^	< 0.01
Heptachlor^a,b^	< 0.01
cis-Heptachlor epoxide^a,b^	< 0.01
trans-Heptachlor epoxide^a,b^	< 0.01
Aldrin^a,b^	< 0.01
Dieldrin^a,b^	< 0.01
Endrin^a,b^	< 0.01
alpha-Endosulfan^a,b^	< 0.01
beta-Endosulfan^a,b^	< 0.01
Methoxychlor^a,b^	< 0.02
2,4ʹ-Dichlorodiphenyldichloroethane (2,4ʹ-DDD)^b^	< 0.01
4,4ʹ-Dichlorodiphenyldichloroethane (4,4ʹ-DDD)^b^	< 0.01
2,4ʹ-Dichlorodiphenyldichloroethylene (2,4ʹ-DDE)^a b^	< 0.01
4,4ʹ-Dichlorodiphenyldichloroethylene (4,4ʹ-DDE)^a,b^	< 0.01
2,4ʹ-Dichlorodiphenyltrichloroethane (2,4ʹ-DDT)^a,b^	< 0.01
4,4ʹ-Dichlorodiphenyltrichloroethane (4,4ʹ-DDT)^a b^	< 0.01
Tecnazene^ab^	< 0.01

Pyrethroids	LOQ (mg/kg)
Instrumentation: GC-ECD (Carlo Erba HRGC 5300)	
lambda-Cyhalothrin^b^	< 0.05
Cypermethrin^b^	< 0.05
Deltamethrin^b^	< 0.05
Fenvalerat^b^	< 0.05
cis-Permethrin^b^	< 0.05
trans-Permethrin^b^	< 0.05
tau-Fluvalinat	< 0.05

^a^Reference pesticides in USP-36; ^b^Reference pesticides in Eu Ph. 9.0

**Table 2:** Organophosphoropesticide and other pesticides screening panel

Organophosphoropesticide and other pesticides			
Instrumentation: GC-ECD (Fisons Instruments HRGC Mega 2 Series)			
	LOQ (mg/kg)		LOQ (mg/kg)
Bromophos-ethyl^a,b^	< 0.02	Fuberidazol	< 0.01
Bromophos-methyl^b^	< 0.02	Furathiocarb	< 0.01
Carbophenothion^b^	< 0.02	Hexaflumuron	< 0.05
cis-Chlorfenvinphos^a^	< 0.05	Hexythiazox	< 0.05
trans-Chlorfenvinphos^a^	< 0.05	Imazalil	< 0.01
Chlorpyrifos-ethyl^a,b^	< 0.02	Imidacloprid	< 0.01
Chlorpyrifos-methyl^a,b^	< 0.02	Indoxacarb	< 0.01
Coumaphos^b^	< 0.05	Ioxynil	< 0.01
Diazinon^ab^	< 0.02	Isoproturon	< 0.01
Dichlofenthion^a,b^	< 0.02	Isoxaben	< 0.01
Ethion^a,b^	< 0.02	Isoxaflutol	< 0.01
Fenchlorphos^a,b^	< 0.02	Kresoxim-methyl	< 0.01
Malathion^a,b^	< 0.02	Linuron	< 0.01
Pirimiphos-ethyl^a,b^	< 0.02	Lufenuron	< 0.01
Phosalone^b^	< 0.05	2-methyl-4-chlorophenoxyacetic acid (MCPA)	< 0.05
Propethamphos^a,b^	< 0.02	4-(4-Chloro-2-methylphenoxy)butanoic acid (MCPB)	< 0.05
Tetrachlorvinphos^a,b^	< 0.02	Mecarbam	< 0.01
Acetamiprid	< 0.01	Mecoprop	< 0.01
Alachlor	< 0.01	Mepanipyrim	< 0.01
Aldicarb	< 0.01	Metalaxyl-M	< 0.01
Ametryn	< 0.01	Metamitron	< 0.01
Amidosulfuron	< 0.01	Methiocarb	< 0.01
Atrazin	< 0.01	Methomyl	< 0.01
Azoxystrobin	< 0.01	Metobromuron	< 0.01
Bendiocarb	< 0.01	Metolachlor	< 0.01
Benfuracarb	< 0.01	Metribuzin	< 0.01
Benomyl	< 0.01	Metsulfuron-metyl	< 0.01
Bensulfuron-methyl	< 0.01	Monolinuron	< 0.01
Bentazon	< 0.01	Monuron	< 0.01
Boscalid	< 0.01	Myclobutanil	< 0.01
Bromoxynil	< 0.01	Nicosulfuron	< 0.01
Buprofezin	< 0.01	Oxadixyl	< 0.01
Carbaryl	< 0.01	Oxamyl	< 0.01
Carbendazim	< 0.01	Oxydemeton-methyl	< 0.01
Carbofuran	< 0.01	Piperonyl butoxide (PBO)	< 0.05
Carboxin	< 0.01	Penconazol	< 0.01
Chloridazon	< 0.01	Pendimethalin	< 0.01
Chlorotoluron	< 0.01	Pirimicarb	< 0.01
Chloroxuron	< 0.01	Promecarb	< 0.01
Chlorsulfuron	< 0.01	Propamocarb	< 0.01
Clethodim	< 0.05	Propargit	< 0.01
Clofentezine	< 0.01	Propoxur	< 0.01
Clomazone	< 0.01	Prosulfocarb	< 0.01
Clothianidin	< 0.01	Prosulfuron	< 0.01
Cyproconazol	< 0.01	Pymetrozin	< 0.01
2,4-dichlorophenoxyacetic acid (2,4-D)	< 0.05	Pyridaben	< 0.01
4-(2,4-dichlorophenoxy)butyric acid (2,4-DB)	< 0.05	Pyrimethanil	< 0.01
Dicamba	< 0.05	Pyriproxyfen	< 0.01
Dichlorprop	< 0.01	Quinmerac	< 0.05
Dicloran	< 0.01	Quizalofop	< 0.01
Diethofencarb	< 0.01	Rimsulfuron	< 0.01
Difenoconazole	< 0.01	Simazin	< 0.01
Diflubenzuron	< 0.01	Spiroxamin	< 0.01
Dinoseb	< 0.01	2,4,5-Trichlorophenoxyacetic acid	< 0.05
Dinoterb	< 0.01	Tebuconazol	< 0.01
Diuron	< 0.01	Tebufenozid	< 0.01
4,6-Dinitro-o-cresol (DNOC)	< 0.01	Tebufenpyrad	< 0.01
Ethoprophos	< 0.01	Teflubenzuron	< 0.01
Etofenprox	< 0.01	Tepraloxydim	< 0.01
Etoxazol	< 0.01	Thiabendazol	< 0.01
Fenamirol	< 0.01	Thiacloprid	< 0.01
Fenazaquin	< 0.01	Thiamethoxam	< 0.01
Fenhexamid	< 0.01	Thifensulfuron-methyl	< 0.01
Fenoxycarb	< 0.01	Triadimefon	< 0.01
Fenpropimorph	< 0.01	Triadimenol	< 0.01
Fenpyroximat	< 0.01	Tribenuron-methyl	< 0.01
Fenuron	< 0.01	Triclopyr	< 0.01
Fipronil	< 0.01	Tridemorph	< 0.01
Florasulam	< 0.01	Triflumizol	< 0.01
Fluazifop-P	< 0.01	Trifloxstrobin	< 0.01
Fluazifop-P-butyl	< 0.01	Triflumuron	< 0.01
Fluazinam	< 0.01	Triflusulfuron	< 0.01
Flufenoxuron	< 0.01	Vamidothion	< 0.01
4-Nonylphenol			< 0.01
4-Octylphenol			< 0.01
4-Nonylphenolethoxylate (mono-, di-)			< 0.01
4-Octylphenolethoxylate (mono-, di-)			< 0.01

^a^Reference pesticides in USP-36; ^b^Reference pesticides in Eu Ph. 9.0

Determination of FLA was performed using the gel permeation chromatographic clean up system followed by chromatographic analysis as described in the USP. Briefly, one gram test lanolin samples were dissolved in methylene chloride eluant, diluted to volume, and filtered. 5.0 mL of the resultant solution was applied to a 25 mm × 100 cm column packed with styrene–divinylbenzene copolymer beads, and eluted with 320 mL eluent, consisting of methylene chloride and hexane (1:1) at a flow rate 4 mL/min. The appropriate fraction was collected in a suitable evaporator and concentrated to 3 mL. This concentrated fraction was then reconstituted in hexane prior to chromatographic analysis according to the methodology outlined in the USP (flow rate 7 mL/min, nitrogen carrier gas, injection volume 1 µL [14] using an Agilent Gas chromatograph 6890 N with MSD 5973 N. Percentage of FLA in each sample was calculated as per the formula described in the USP monograph for modified lanolin.

Acid value was determined as per the method described in the European Pharmacopoeia wool fat monograph [20]. Briefly, 5 g test lanolin samples were dissolved in 25 mL of a mixture of equal volumes of previously neutralised ethanol and light petroleum, with phenolphthalein solution R1 included as an indicator. Once dissolved, the sample was titrated with 0.1 M potassium hydroxide until the pink colour persists for at least 15 s (n mL of titrant), indicating complete neutralisation has occurred. The acid value is the number that expresses, in milligrams, the quantity of potassium hydroxide required to neutralise the free acids present in 1 g of the test substance and can have a maximum value of 1.0 [20].

Peroxide value was determined as per the method described in the European Pharmacopoeia wool fat monograph [20]. Briefly, 5 g test lanolin samples were dissolved in 30 mL of a mixture of chloroform and glacial acetic acid. Once dissolved, 0.5 mL of saturated potassium iodide solution was added and the mixture was shaken for 1 min exactly. Thirty millilitres of water were then added and the resultant solution titrated with 0.01 M sodium thiosulfate. The peroxide value is the number that expresses in milliequivalents of active oxygen, the quantity of peroxide contained in 1000 g of the test substance and is calculated as per the formula outlined in the Ph. Eur. 9.0, compared to a blank titration under the same conditions, with a maximum value of 20.0.

Anisidine value was determined as per the method described in the European Pharmacopoeia 9.0. Briefly, 0.5 g of the test lanolin samples were dissolved in trimethylpentane R and diluted to 25.0 mL in the same solvent (test solution A). A second test solution (B) was also prepared by mixing 5.0 mL of test solution A with 1.0 mL of a 2.5 g/L solution of p-anisidine prepared in glacial acetic acid, and the absorbance measured after 10 min. The anisidine value is defined as 100 times the optical density measured in a 1 cm cell of a solution containing 1 g of the substance to be examined in 100 ml of a mixture prepared as per the above method and is calculated as per the formula outlined in the Ph. Eur. 9.0, compared to a reference standard of p-anisidine alone and absorbance values for test solutions A and B [20].

### Statistical analysis

Data from the sample analysis was tabulated and mean averages calculated in instances where an analyte was detected in multiple replicates. Standard deviations were also determined. In cases where an analyte was present in only one replicate but below detection limits in others, the mean and standard deviation were not calculated. There are a several methods which can be applied to statistically analyse data containing non-detects, including simple substitution, the Kaplan–Meier method and Regression Order Statistics [21]. However, the small number of replicates in this study mean that the application of these methods was considered inappropriate with the potential to misrepresent the results, particularly as many residue values were extremely close to detection limits. Where appropriate, the significance of any differences was calculated by two tailed T-test, with a p-value < 0.05 considered significant.

## Results

### Pesticide residue analysis

None of the 21 OCP included in the screening were detected in any of the lanolin product or ingredient samples. However, trace residues of the OPP Diazinon were detected in all three PURELAN samples (average concentration detected 0.127 mg/kg; stdev 0.125) (Table 3). Two out of three PURELAN samples also contained a second pesticide, Piperonyl butoxide (PBO) (average concentration 0.185 mg/kg; stdev 0.120). Diflubenzuron (0.02 mg/kg), Triflumuron (0.02 mg/kg), and the pyrethroid Cypermethrin (0.09 mg/kg) were detected in single PURELAN replicates, with the other two samples determined to be below detection limits (Table 3). The single test sample of the CORONA-8 ingredient was found to contain residues of several OPP and other pesticides; Chlorpyrifos-ethyl (1.50 mg/kg), Diazinon (0.69 mg/kg) Ethion (0.27 mg/kg) and PBO (1.30 mg/kg), however no pyrethroids were detected. No pesticide residues of any kind were detected in the HPA LANOLIN or PHARMALAN ingredient samples, with all values below the limits of detection (Table 3).

**Table 3:** Summary: detected pesticide residues, Free Lanolin Alcohols, and Oxidation metabolites

Product	HPA LANOLIN			PURELAN			PHARMALAN	CORONA-8
LOT:	13099	17303	14355	011161–5	006222–6	004231–1	0001739569	0001572904
Free Lanolin Alcohols (FLA) (%)	0.49	0.56	0.79	2.80	2.50	3.00	1.24	4.50
Average FLA (%) *(S.D.)*	**0.61** (0.16)*			**2.78** *(0.25)*			n/a	n/a
Oxidation Metabolites (value)								
Peroxide Value (PV)	6.10	5.70	8.00	14.00	11.00	12.90	11.00	12.00
Average PV *(S.D.)*	**6.60** (1.23)*			**12.63** *(1.52)*			n/a	n/a
Acid Value (AV)	0.84	0.67	0.67	0.39	0.38	0.43	0.90*	0.50
Average AV *(S.D.)*	**0.73** *(0.10)*			**0.40** *(0.03)*			n/a	n/a
Anisidin Value (AnV)	2.20	1.00	5.30	5.70	6.90	12.90	5.60	3.30
Average AnV *(S.D.)*	**2.83** *(2.22)*			**8.50** *(3.86)*			n/a	n/a
Pyrethroids (mg/kg)								
Cypermethrin	< 0.05	< 0.05	< 0.05	< 0.05	< 0.05	**0.09**	< 0.05	< 0.05
Organophosphoropesticides (OPP) (mg/kg)								
Chlorpyrifos-ethyl	< 0.02	< 0.02	< 0.02	< 0.02	< 0.02	< 0.02	< 0.02	**1.50**
Diazinon	< 0.02	< 0.02	< 0.01	**0.27**	**0.04**	**0.07**	< 0.02	**0.69**
				**0.127** *(0.125)*				
Diflubenzuron	< 0.01	< 0.01	< 0.01	< 0.01	**0.02**	< 0.01	< 0.01	< 0.01
Ethion	< 0.02	< 0.02	< 0.02	< 0.02	< 0.02	< 0.02	< 0.02	**0.27**
PBO	< 0.05	< 0.05	< 0.05	< 0.05	**0.10**	**0.27**	< 0.05	**1.30**
				**0.185** *(0.120)*				
Triflumuron	< 0.01	< 0.01	< 0.01	**0.02**	< 0.01	< 0.01	< 0.01	< 0.01
Total pesticide residues (mg/kg)	n/a	n/a	n/a	**0.29**	**0.16**	**0.34**	n/a	**3.76**
				**0.263** *(0.09)*				

*Significant difference

### Free LANOLIN alcohol analysis

FLA were detected and quantified in all test samples. The HPA LANOLIN had the lowest FLA concentration (average 0.61%, stdev 0.16) while the PHARMALAN and the CORONA-8 had a FLA concentration of 1.24% and 4.50% respectively. The average FLA concentration of PURELAN was 2.78% (stdev 0.25) (Table 3). FLA levels in the HPA LANOLIN were significantly lower than the PURELAN samples (p =  < 0.00023).

### Oxidation metabolites analysis

The Peroxide value of the HPA LANOLIN samples averaged 6.60 (stdev 1.23). The PURELAN, CORONA-8 and PHARMALAN samples were comparable in terms of peroxide value; reporting values of 12.63 (stdev 1.52), 12.00 and 11.00 respectively (Table 3). The peroxide value of the PURELAN samples was significantly higher than the HPA LANOLIN (p = 0.005). Acid values for all samples tested were as follows; HPA LANOLIN average 0.73 (stdev 0.10), PURELAN average 0.40 (stdev 0.03), CORONA-8 0.50, PHARMALAN 0.90. The difference in acid value between the PURELAN sample and the HPA LANOLIN and PHARMALAN was found to have significance (p =  < 0.05) Anisidine value also varied between test materials, but differences were not significant, with HPA LANOLIN average 2.83 (stdev 2.22), PURELAN average 8.50 (stdev 3.86), CORONA-8 value 3.30 and PHARMALAN value 5.60 (Table 3).

## Discussion

Pesticides have been used extensively in modern farming and agricultural practice, to protect crops against insects, weeds and other pests [22]. They are also used directly on the fleece of sheep to prevent mite infestation [23]. However, the negative impact of pesticides for both humans and the environment has become increasingly apparent. Many older generation pesticides can persist for years in the environment, meaning that residues are commonly detected in food and natural cosmetic ingredients [23–26]. There is a global drive to reduce unnecessary pesticide use, bolstered in part by an increased consumer preference in western markets for organic products [27]. However, pesticides still play a significant role in food production in many countries and therefore contamination of sheep’s wool and lanolin is still a concern [25, 26, 28, 29].

The extensive panel of pesticides included in this screening was based on those listed as reference pesticides in the ‘Pesticide Residues Impacting Wool Fat’ in the European Pharmacopoeia and within the United States Pharmacopeia’s Monograph for Modified Lanolin [14, 15, 20] but was expanded to include other pesticides not listed within these documents but known to be possible contaminants of lanolin or other natural materials. The panel included three major classifications of pesticides. OCP have been used on a huge scale over the last century but are now banned globally. However, their long-term persistence means that contamination of plants and entry into the food chain through consumption by livestock is still a real issue [28].

The second class of pesticides included in the panel, OPP, are still widely used for agricultural, veterinary and residential applications, however control of their use is increasing. In the past decade, several notable OPP have been discontinued for use, including parathion, which is no longer registered for any use, and chlorpyrifos, which is no longer registered for home use [30]. OPP can be rapidly degraded by hydrolysis in sunlight, air, and soil and biodegraded by soil bacteria, but small amounts may persist in food and drinking water [31]. Despite their ubiquitous use, the World Health Organization (WHO) has classified OPP as extremely dangerous chemical compounds [32]. Pyrethroid insecticides were also included in the panel. These chemicals have become popular replacements as older OCP were phased out for environmental and human health reasons. Pyrethroids are widely used in public health because of their relative safety for humans, high insecticidal potency at low dosages and rapid effects, and while they appear to have less associated toxicity issues than older insecticides, recommendation is still that levels of exposure should be kept to a minimum [29].

Despite the broad panel of pesticides included in the screening, and the high sensitivity of the assays which enabled even trace residues present at ppm concentrations to be detected, the majority of the pesticides were not detected in any of the lanolin samples tested. Where pesticide residues were detected, they were present at levels close to the limits of quantification. This reflects the fact that all test materials included in the study were highly refined. While published investigations of pesticide residues in wool wax and lanolin in recent decades is limited, an analysis of Uruguayan lanolin samples by Perez et al. reported a similar residue profile to the samples tested here; with residues of chlorpyriphos, cypermethrin, diazinon and ethion identified [33]. A second study, which screened for 40 OPP residues in raw sheep wool from Uruguay, detected only diazinon and ethion residues [34]. The quantification of pesticide residues from other cosmetic ingredients, such as citrus essential oils and beeswax suggests pesticide contamination of these materials is higher than is seen for lanolin, unsurprising given the refinement process that converts wool grease into the final lanolin material [33]. As an example, one study of Spanish commercial beeswax found it to contain chlorimefon, chlorfenvinphos, chlorpyriphos, endosulfan and malathion residues [35]. Of over 197 samples analysed, the authors reported residues of chlorfenvinphos residues in 96% of samples with concentrations up to 10.6 mg/kg residues [35]. Similarly, a study by Mullin et al. found that almost 98% of the North American beeswax samples tested were contaminated with up to 204 and 94 mg/kg residues of τ-fluvalinate and coumaphos respectively [36]. With these high values in mind, the pesticide residues detected in the refined lanolin evaluated in this study are more comparable to residual pesticide levels of edible oils. Parrilla Vázquez et al. analysed 17 different Spanish olive oil, soyabean oil and sunflower oil samples, detecting 14 different pesticides across 10 of the refined samples, at concentrations between 0.012 and 0.156 mg/kg [37]. A study on Chinese edible oils by Jing et al. yielded similar values [38]. This similarity to edible oils gives further confidence that incidental ingestion of lanolin by the nursing infant should not be a concern.

However, pesticide residue content did differ between the test samples. When considering the results for the two lanolin ingredients included in the study, PHARMALAN and CORONA-8, it should be noted that they were different grades of lanolin and therefore will have undergone different levels of refinement and purification to remove contaminants during their manufacture. PHARMALAN is a purified grade of anhydrous lanolin, manufactured for dermatology and baby care indications to a specification in compliance with the European Pharmacopoeia standard. The CORONA-8 lanolin is a cosmetic grade of lanolin suitable for inclusion in skin care, soaps and lipstick products, however it is not a monograph compliant ingredient. Comparison of the residue analysis data for these two samples demonstrated the impact of extra refinement on lanolin purity. The PHARMALAN ingredient contained no detectable pesticide residues of any kind, meaning its purity exceeded that outlined in the Ph. Eur. specification. Interestingly, although the CORONA-8 material was not a monograph compliant ingredient, it only contained trace residues of 4 individual pesticides of the total 178 included in the screening. However, three of the four pesticide residues present in the CORONA-8 sample were specified in both the USP and Ph. Eur. monographs and were present at concentrations in excess of those permitted by these standards. The FLA content of PHARMALAN also reflected its increased refinement; it contained only 1.24% free lanolin alcohols compared to 4.50% present in the CORONA-8 sample (Table 3).

The two lanolin products included in the study also differed from each other. The HPA LANOLIN did not contain any detectable pesticide residues, therefore exceeding the requirements for a lanolin compliant with the Ph. Eur. and USP Monographs and consistent with the residue analysis of PHARMALAN. The second nipple care lanolin tested, PURELAN, was found to contain a number of pesticide residues, some of which are specified within the monograph. The levels of pesticide residues detected were within the permitted limits and so despite their presence, the PURELAN was still compliant with the USP and Ph. Eur. monographs and was superior in purity to the cosmetic grade CORONA-8 ingredient. It was, however, inferior in purity to the PHARMALAN ingredient material and HPA LANOLIN (Table 3).

It was notable that the average percentage of FLA in the HPA LANOLIN was extremely low (0.61%); half the amount present in the PHARMALAN ingredient, and more than fourfold less than the average FLA content for PURELAN. It has previously been demonstrated that reducing the FLA content of lanolin reduces its sensitising potential, and furthermore, that reduction of FLA to below 1.5% results in a hypoallergenic material when tested on patients with pre-existing lanolin sensitivity [13]. With this context, and given that the intended use of the test lanolin products is repeated and frequent application to the nipples of breastfeeding mothers to relieve soreness, the extremely low FLA content of the HPA LANOLIN, achieved through ultra-refinement, may offer an advantage over the other test samples in minimising the risk of allergy or adverse reaction in the user.

The anisidine, acid and peroxide values of all test samples were similar, although again, the HPA LANOLIN reported the lowest values. These measures of stability are especially important for lanolin intended for use in nipple care; a high value for these tests indicates increased rancidity which can negatively impact taste and smell of the material. The results for all lanolin samples tested indicate a good quality material with levels well within acceptable limits. This should result in a neutral and odourless material which is important for any product used on nipples during breastfeeding to prevent interference with nursing.

Due to the nature of the methods used, there were limitations on the number of samples which could be tested. Triplicate samples from three individual batches were tested for the two lanolin products, while the lanolin ingredients were only tested in singlicate. This was due to difficulties in obtaining multiple batches of the raw material ingredients. Some inter-batch variability in pesticide residue content was seen for the lanolin products, which makes sense given the intrinsic variability of the raw material. We acknowledge that such variability would likely also have been apparent for the PHARMALAN and CORONA-8 samples had multiple replicates been tested. However, as the lanolin ingredients were included to provide a general benchmark of refinement rather than being the focus of the study, an n = 1 for these materials was considered acceptable without compromising study integrity.

## Conclusions

In conclusion, it is critical that any lanolin intended for use on the nipples of breastfeeding women be as pure as possible. Increasing consumer awareness around ingredient origin, ‘clean’, pesticide-free and organic products may feasibly raise questions around pesticide contamination in nipple creams, particularly as there will be some level of incidental infant exposure to the product during use. The data presented here supports the safety of such products with regards to pesticide contamination; comparison to the raw material ingredients revealed the positive impact of refinement on pesticide residue levels, while the results for the lanolin nipple creams demonstrates that both products exceeded the purity requirements set by the Ph. Eur. and USP monographs confirming they are appropriate for their intended use. The modern refinement processes applied to lanolin means that historic associations with allergy and sensitisation no longer apply, however for users where allergenicity may be a consideration, the data presented here indicates that HPA LANOLIN is particularly suitable due to its extremely low free lanolin alcohol content.

## Data Availability

Test reports for the independent residue analysis are archived and available upon request, please email the corresponding author, katie@lansinoh.co.uk.

## Abbreviations

FLA: Free lanolin alcohols
USP: United States Pharmacopoeia
Ph. Eur.: European Pharmacopoeia
OCP: Organochloropesticides
OPP: Organophosphoropesticides
Ppm: Parts per million
AV: Acid value
PV: Peroxide value
AnV: Anisidine value
WHO: World Health Organisation
